# Distance Learning and Spaced Review to Complement Dermoscopy Training for Primary Care

**DOI:** 10.5826/dpc.1102a30

**Published:** 2021-04-12

**Authors:** Elizabeth V. Seiverling, Danielle Li, Kathryn Stevens, Peggy Cyr, Gregory Dorr, Hadjh Ahrns

**Affiliations:** 1 Dermatology Division, Maine Medical Center, Portland, Maine, USA; 2 Tufts University School of Medicine, Boston, USA; 3 Maine Medical Partners Family Medicine, Portland, USA; 4 Maine Medical Center Quality Improvement, Portland, USA

**Keywords:** dermoscopy, primary care, skin cancer, distance learning, resident education

## Abstract

**Background:**

Dermoscopy aids in skin cancer identification. For family physicians who use dermoscopy, there is higher sensitivity for melanoma detection than naked-eye examination. There is a shortage of dermoscopy training for primary care providers. The triage amalgamated dermoscopic algorithm (TADA) is designed for novice dermoscopists. While TADA can be taught in a short dermoscopy workshop, spaced review and blended learning strategies improve knowledge retention.

**Objectives:**

This study determined the impact that the addition of a distance learning platform has on clinical dermoscopy use. Moreover, it evaluated dermoscopic image identification (knowledge retention) following the addition of distance learning via Extension for Community Health Outcomes (ECHO) to a traditional TADA dermoscopy workshop.

**Methods:**

Primary care providers voluntarily attended a 120-minute TADA dermoscopy workshop. Participants completed pre-intervention, post-TADA, and post-ECHO tests of 30 dermoscopic images of benign and malignant skin lesions. A survey was also administered to analyze clinical dermoscopy use and prior dermoscopy training.

**Results:**

Twenty-seven residents, faculty, and advanced practice providers participated in this longitudinal observational cohort study. Mean test scores (out of 30) for images of benign and malignant lesions improved from 20.29 pre-intervention to 24.62 post-TADA and 27.63 post-ECHO (P < .001). On average, participants attended 4 ECHO sessions (out of 7 total) and there was a positive correlation (*r* = 0.77) between the number of ECHOs attended and post-ECHO scores. Dermoscope use increased from 37.0% to 96.3% (P < .001).

**Conclusion:**

Distance learning and spaced review complement dermoscopy workshop training for primary care.

## Introduction

Skin cancer affects 1 in 5 Americans [1], with melanoma accounting for 10,000 deaths annually in the United States [2]. Maine has the sixth highest incidence of melanoma in the U.S. [3]. However, when melanoma is detected early, 5-year survival rates are 98% [2]. In the U.S., there is a nationwide shortage of dermatologists, with rural states, such as Maine, most profoundly affected. Maine has one of the lowest ratios of dermatologists per 100,000 people [4]. Due to a shortage of dermatologists, skin cancer is often detected by primary care providers (PCPs). Indeed, many patients seek dermatologic care from their PCPs, and 12%–25% of primary care visits are due to a dermatologic concern [5,6]. Thus, PCPs are often the first medical providers approached when a patient has a skin growth of concern.

Dermoscopy is a useful and cost-effective tool for melanoma detection in primary care [7]. Dermoscopes are hand-held instruments that use polarized and non-polarized light to illuminate subsurface skin structures (epidermis, dermoepidermal junction, and papillary dermis). Most of these structures cannot otherwise be seen with the naked eye. For family physicians who use dermoscopy, there is higher sensitivity for melanoma detection than naked-eye examination [7]. However, the majority of dermoscopy training has been geared toward dermatologists, not PCPs. There is no consensus on how best to teach dermoscopy. Furthermore, there is no agreement on whether or not dermoscopy training should be the same for dermatologists and PCPs.

Previous research has identified the triage amalgamated dermoscopic algorithm (TADA) as an effective option for training primary care [8,9]. TADA is a simplified dermoscopy algorithm with high sensitivity and specificity for benign and malignant growths [8,9]. Following training with TADA, the sensitivity for dermoscopic identification of malignant skin growths increased from 62.5% to 88.1% in family physicians. The specificity for dermoscopic identification of common benign growths (seborrheic keratosis, dermatofibroma, angioma) was over 90% [8].

The durability of TADA training is not known. However, spaced review and blended learning strategies are well-established teaching modalities to aid with knowledge retention [12–14]. To incorporate alternative learning strategies and spaced review into our dermoscopy training, we added a distance learning platform. Distance learning, also termed distance education, is a form of education in which courses are delivered via the Internet without face-to-face interaction between student and instructor [12,15]. Project Extension for Community Health Outcomes (ECHO) is a distance learning platform used in medicine to create learning loops between specialists and PCPs.

Project ECHO uses videoconferencing technology to link students, residents, and practicing providers with specialists, for real-time learning and clinical practice support. The goal is to improve knowledge at the local level, resulting in fewer unnecessary referrals and better access to care. Because of the ongoing (monthly) nature of Project ECHO, participants have an opportunity to build on initial dermoscopy training. Furthermore, critical to Project ECHO is case presentation and spaced review, which is essential to creating long-term memory [13,14].

The specific objectives of this project were to:

- Determine the impact the addition of a distance learning platform has on clinical dermoscopy use.
- Evaluate dermoscopic image identification (knowledge retention) following the addition of distance learning via Project ECHO to a traditional TADA dermoscopy workshop.

## Methods

This project was reviewed by the Maine Medical Center [MMC] Institutional Review Board and was deemed exempt. Participants were recruited via email invitation. Participation was voluntary and involved a single, in-person, 120-minute TADA dermoscopy workshop without compensation. Participants were granted hours towards their continuing medical education requirement. To be eligible, participants had to be: [1] a primary care provider (resident, faculty, or an advanced practice provider (APP) in Family Medicine or Internal Medicine), and [2] an employee of MMC or MaineHealth.

A pre-intervention dermoscopy test and survey was administered prior to the TADA dermoscopy workshop. The test contained 30 dermoscopic images of benign and malignant neoplasms. The survey addressed demographics and asked questions regarding dermoscopy use. Immediately following the TADA dermoscopy workshop, participants were asked to complete a post-TADA test with a different set of 30 dermoscopic images. None of the images in the post-TADA test were used during the educational workshop. The dermoscopy images were selected from MaineHealth’s teaching database and had been collected by author E.V.S. at MMC. Images were reviewed by E.V.S., H.T.A., and K.M.S. Ninety images were deemed representative examples of benign and malignant neoplasms. These 90 images were then utilized in the pre-intervention, post-TADA and post-ECHO tests.

Upon completion of a TADA workshop, participants were then eligible to take part in longitudinal dermoscopy training via Project ECHO. Seven dermatology/dermoscopy ECHOs were offered over the course of 8 months. Each ECHO adhered to a standard 60-minute format with a 20- to 30-minute didactic lecture followed by interactive cases. The topics for the didactic portion included the following: basal cell carcinoma, nevus versus melanoma, psoriasis and other scaly rashes, common skin infections, dermoscopy of benign skin tumors, skin biopsy techniques, and a dermoscopy recapitulation.

At the conclusion of Project ECHO, participants took a post-ECHO test consisting of 30 additional dermoscopic images of benign and malignant growths. In addition to the dermoscopic image test, survey questions addressing frequency of dermoscopy use were also administered. Descriptive comparisons of scores were made using mean scores along with standard deviations and were compared using the Wilcoxon signed-rank test.

## Results

This longitudinal observational cohort study conducted in the State of Maine included 27 residents, faculty, and APPs from MMC and MaineHealth. Table 1 lists the characteristics of the 27 participants included in the analysis. This sample was predominantly female (70.4%, n = 19), with MD or DO qualifications (96.3%, n = 26), working as a family physician (88.9%, n = 24), and with fewer than 10 years of experience evaluating skin lesions (66.7%, n = 18). Only 11.1% of participants had formal training in dermoscopy prior to the study.

Table 2 highlights survey questions that participants answered pre- and post-intervention. All participants reported access to a dermoscope post-intervention. Pre- TADA and post-ECHO surveys showed that dermoscope use increased from 37.0% to 96.3% (chi-square test, P < .001), with the majority of participants now using a dermoscope weekly.

Overall, mean test scores (out of 30) for images of benign and malignant neoplasms improved from 20.29 pre- intervention to 24.62 post-TADA and 27.63 post-ECHO.

**Figure 2:**
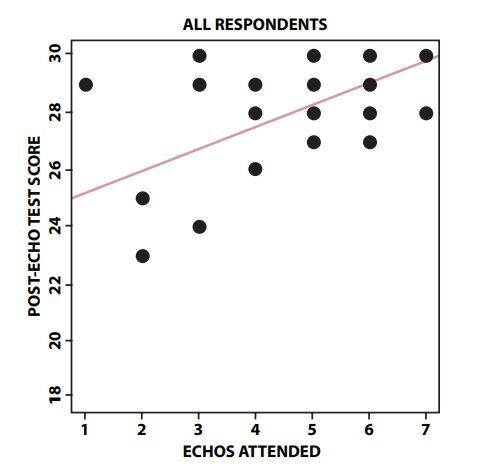
Linear regression of participant Extension for Community Outcomes (ECHO) attendance and post-ECHO test scores. Some dots represent multiple participants with the same test score and ECHO attendance.

On average, participants attended 4 ECHO sessions out of 7 total. Figure 2 highlights the results of a linear regression that shows a positive correlation (*r* = 0.77) between the number of ECHO sessions attended and post-ECHO test scores.

## Discussion

While dermoscopy aids in skin cancer detection and the American Academy of Family Physicians recommends all Family Medicine residents be trained in dermoscopy, there is no standard dermoscopy training for PCPs [16]. To improve dermoscopy training for primary care in our health system, we added monthly dermoscopy training sessions via Project ECHO. This dermoscopy-focused ECHO was the first geared toward primary care in the U.S. Based on our results, distance learning platforms such as Project ECHO might be viable options for ongoing dermoscopy training for PCPs. The monthly format of Project ECHO allows for spaced review, and in this study, led to knowledge retention. Improved image identification in the classroom setting does not necessarily translate to increased dermoscopy use in the clinical setting, yet, our study shows that participants dramatically increased the use of dermoscopy in their practices.

While the participants in our study reported the most beneficial way to learn dermoscopy was with the live TADA workshop and in-person clinical rotations, remote session learning through Project ECHO was also a preferred learning modality. Live dermoscopy workshops and in-clinic training with a dermoscopist is not feasible at many primary care sites. However, distance learning allows for virtual consults and telementoring that can serve as a surrogate to physical time in the clinic with a dermoscopy expert [17]. Telementoring allows learners at multiple sites to access dermoscopy training at the same point in time.

Concurrent with the implementation of the multimodal dermoscopy curriculum (which included the TADA workshops, clinical dermatology rotations and ECHO), a dermatology electronic consultation (eConsult) platform was launched within our health system. This allowed participants to apply their dermoscopy training to real cases and ask for expert opinion on their own patients. The synchronous introduction of eConsult and Project ECHO may have contributed to increased dermoscope use.

Distance learning through Project ECHO may provide a solution to effectively facilitating dermoscopy training and virtual consults in rural settings. While this study was initiated prior to the onset of COVID-19, the pandemic has emphasized the importance of adding distance learning to medical education. We hope this innovative approach to teaching dermoscopy to PCPs will motivate other institutions to consider distance learning platforms and spaced review to complement dermoscopy workshops and “boot camps.”

This study was limited by its sample size. Due to disruption caused by COVID-19, there was a poor retention rate. Many of our participants were deployed to inpatient services and unable to complete the final quiz. The initial TADA workshops were attended by over 100 clinicians. We have only included participants who completed all 3 tests in this report.

## Figures and Tables

**Figure 1:**
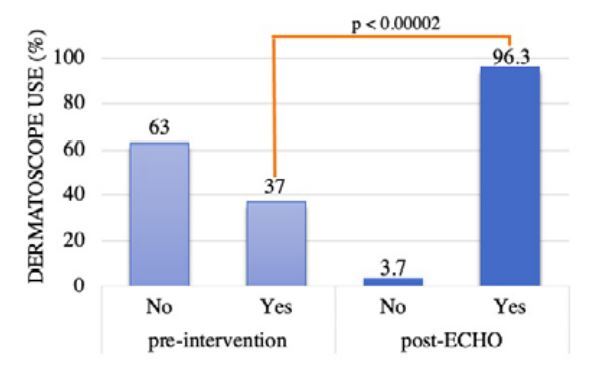
Pre-intervention and Post-ECHO dermoscopy use

**Table 1:** Characteristics of 27 Study Participants^a^

Variable	Coding	No. (%)
Overall		27 (100.0)
Sex	Male	8 (29.6)
	Female	19 (70.4)
Age	21–30 y	13 (48.1)
	31–40 y	9 (33.3)
	41–50 y	3 (11.1)
	51–60 y	2 (7.4)
Training	MD/DO	26 (96.3)
	Board-Certified PCP	9 (33.3)
	Resident	17 (63.0)
	Nurse Practitioner	1 (3.7)
Specialty	Family Medicine	24 (88.9)
	Internal Medicine	3 (11.1)
Years evaluating skin lesions	≤10	18 (66.7)
	10+	3 (11.1)
Any formal training in dermoscopy?	No	23 (85.2)
	Yes	3 (11.1)

a Not all participants answered every question on the survey.

PCP = primary care provider.

**Table 2:** Pre- vs. Post-Intervention Survey Results for 27 Participants a

Variable	Coding	Pre-intervention, No. (%)	Post-intervention, No. (%)
Do you have access to a dermoscope?	No	7	(26.0) 0 (0)
	Yes	20 (74.0)	27 (100)
Do you use a dermoscope?	No	16 (59.3)	1 (3.7)
	Yes	10 (37.0)	26 (96.3)
If yes, how often?	Daily	0 (0.0)	1 (3.7)
	2–3×/week	1 (3.7)	4 (14.8)
	1×/week	1 (3.7)	9 (33.3)
	2–3×/month	2 (7.4)	5 (18.5)
	1×/month	3 (11.1)	5 (18.5)
	<1×/month	3 (11.1)	2 (7.4)

a Not all participants answered every question on the survey.

## References

[1] Kerr OA, Tidman MJ, Walker JJ, Aldridge RD, Benton EC (2010). The profile of dermatological problems in primary care. Clin Exp Dermatol.

[2] Cancer facts & figures (2017). American Cancer Society.

[3] Rate of new cancers in the United States, melanomas of the skin.

[4] Glazer AM, Farberg AS, Winklemann RR, Rigel DS (2017). Analysis of trends in geographic distribution and density of US dermatologists. JAMA Dermatol.

[5] Perera E, Xu C, Manoharan S, Soyer HP, Binder M, Smith A, Wurm E (2011). Real-life teledermatology cases. Telemedicine in Dermatology.

[6] Verhoeven EW, Kraaimaat FW, van Weel C (2008). Skin diseases in family medicine: prevalence and health care use. Ann Fam Med.

[7] Herschorn A (2012). Dermoscopy for melanoma detection in family practice. Can Fam Physician.

[8] Seiverling EV, Ahrns HT, Greene A (2019). Teaching benign skin lesions as a strategy to improve the triage amalgamated dermoscopic algorithm (TADA). J Am Board Fam Med.

[9] Susong JR, Ahrns HT, Daugherty A, Marghoob AA, Seiverling EV (2020). Evaluation of a virtual basic dermatology curriculum for dermoscopy by using the triage amalgamated dermoscopic algorithm for novice dermoscopists. J Am Acad Dermatol.

[10] Wu TP, Newlove T, Smith L, Vuong CH, Stein JA, Polsky D (2013). The importance of dedicated dermoscopy training during residency: A survey of US dermatology chief residents. J Am Acad Dermatol.

[11] Chen YA, Rill J, Seiverling EV (2017). Analysis of dermoscopy teaching modalities in United States dermatology residency programs. Dermatol Pract Concept.

[12] Ruiz JG, Mintzer MJ, Leipzig RM (2006). The impact of E-learning in medical education. Acad Med.

[13] Augustin M (2014). How to learn effectively in medical school: Test yourself, learn actively, and repeat in intervals. Yale J Biol Med.

[14] Khajah MM, Lindsey RV, Mozer MC (2014). Maximizing students’ retention via spaced review: practical guidance from computational models of memory. Top Cogn Sci.

[15] Distance education.

[16] Family medicine residency curriculum guidelines: Conditions of the skin.

[17] Nelson KC, Gershenwald JE, Savory SA (2019). Telementoring and smartphone-based answering systems to optimize dermatology resident dermoscopy education. J Am Acad Dermatol.

